
JOURNAL INFORMATION
==============================
NLM Title Abbreviation: Biospektrum (Heidelb)
Journal ID: Biospektrum (Heidelb)
NLM Journal ID: 9615685

Biospektrum

ISSN: 0947-0867
EISSN: 1868-6249

ARTICLE INFORMATION
==============================
PMCID: PMC8111370
PMID: 33994672
DOI: 10.1007/s12268-021-1564-x
Article ID: 1564
Article version: 1
Subjects: Wissenschaft

Impfstoffe für alle? Dodecin als Impfstoffplattform

Bourdeaux Florian bourdeaux@chemie.uni-frankfurt.de
1
Rittner Alexander rittner@chemie.uni-frankfurt.de
1
Grininger Martin 1 2
1 grid.7839.5 0000 0004 1936 9721 Institut für Organische Chemie und Chemische Biologie, Goethe Universität Frankfurt, Max-von-Laue-Straße 7, D-60438 Frankfurt a. M., Deutschland
2 grid.7839.5 0000 0004 1936 9721 Buchmann Institut für Molekulare Lebenswissenschaften, Universität Frankfurt a. M., Frankfurt, Deutschland
Electronic publication date: 2021 May 11
Print publication date: 2021
Volume: 27
Issue: 3
First page: 250
Last page: 253
Copyright: © Die Autoren 2021
License: Dieser Artikel wird unter der Creative Commons Namensnennung 4.0 International Lizenz veröffentlicht, welche die Nutzung, Vervielfältigung, Bearbeitung, Verbreitung und Wiedergabe in jeglichem Medium und Format erlaubt, sofern Sie den/die ursprünglichen Autor(en) und die Quelle ordnungsgemäß nennen, einen Link zur Creative Commons Lizenz beifügen und angeben, ob Änderungen vorgenommen wurden. Die in diesem Artikel enthaltenen Bilder und sonstiges Drittmaterial unterliegen ebenfalls der genannten Creative Commons Lizenz, sofern sich aus der Abbildungslegende nichts anderes ergibt. Sofern das betreffende Material nicht unter der genannten Creative Commons Lizenz steht und die betreffende Handlung nicht nach gesetzlichen Vorschriften erlaubt ist, ist für die oben aufgeführten Weiterverwendungen des Materials die Einwilligung des jeweiligen Rechteinhabers einzuholen. Weitere Details zur Lizenz entnehmen Sie bitte der Lizenzinformation auf http://creativecommons.org/licenses/by/4.0/deed.de.
License URL: https://creativecommons.org/licenses/by/4.0/

issue-copyright-statement: © Springer-Verlag GmbH Deutschland, ein Teil von Springer Nature 2021

==============================
The Corona pandemic has painfully taught us the threat of new pathogens in a globalized world and how vital modern vaccines are. Platform technologies play an important role in the discovery of new vaccines as reducing the time for the development dramatically — time that saves lives. Here, we present the protein Dodecin and how it may be utilized as a versatile platform technology to produce cheap and robust new vaccines for everyone in all parts of the world.

■ Der Antagonismus von Pathogenen und Wirten ist eine wesentliche treibende Kraft in der Entstehung und Evolution von Leben. Dabei erscheint das ewige Wettrennen mit viralen oder mikrobiellen Krankheitserregern auf den ersten Blick für höher entwickelte Wirte wie den Menschen geradezu aussichtslos [1] – wie sollen sie mit Pathogenen koexistieren, deren Evolutionsrate 10000-mal schneller ist als die ihrige? Die Antwort ist ein ausgeklügelter Abwehrmechanismus, der nicht den Generationenwechsel zur Anpassung nutzt, sondern mit der immensen Mutationsrate von Pathogenen Schritt halten kann: das adaptive Immunsystem [1].

Hocheffizient, bringt es dennoch ein großes Manko mit sich: Es braucht Zeit, um auf eine Infektion zu reagieren; Zeit, in der sich Pathogene im Körper ausbreiten können. Aus diesem Grund werden bereits seit dem 18. Jahrhundert Hilfsmittel entwickelt, die das Gleichgewicht zugunsten des Immunsystems verschieben sollen: die Vakzine. Sie verkürzen das Zeitfenster, in dem sie dem Immunsystem bereits vor der Infektion die nötigen Informationen zur Bekämpfung bereitstellen.

Dabei basieren die Informationen in modernen Vakzinen nicht mehr auf vollständigen Pathogenen, sondern auf winzigen, aber spezifischen pathogenen Schlüsselbestandteilen, die hier vereinfacht Antigene genannt werden. Der große Vorteil beim Verwenden von Antigenen ist, dass sie keine Infektion im Körper der Geimpften auslösen können, dort aber zur Produktion neutralisierender Antikörper führen, sodass ein Pathogen nach Eintritt unter Mithilfe von Immunzellen unschädlich gemacht wird.

## Moderne Vakzine – Impfstoffe für alle?

Aktuell eindrucksvollstes Beispiel ist die Entwicklung moderner Vakzine gegen das SARS-CoV-2-Virus im Kampf gegen die

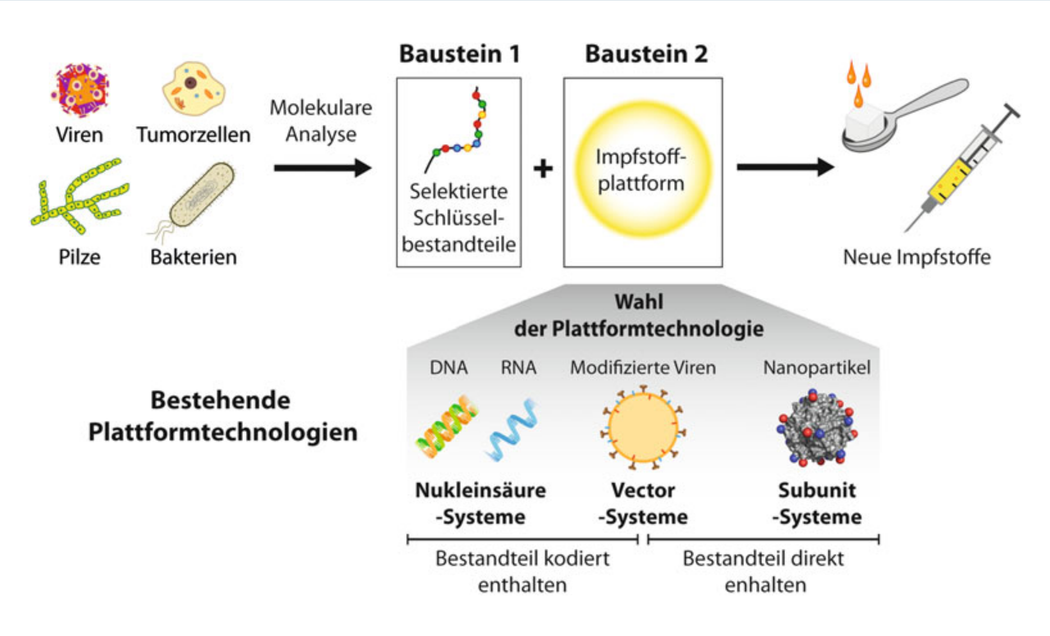

◀ **Abb. 1:** Baukasten für moderne Impfstoffe. Im ersten Schritt wird ein Schlüsselbestandteil bzw. werden mehrere Schlüsselbestandteile des Pathogens ausgewählt, hier beispielhaft Viren, Tumorzellen, Pilze und Bakterien. Im zweiten Schritt werden die Schlüsselbestandteile mit einer Impfstoffplattformtechnologie kombiniert, wodurch sie codiert oder direkt vorliegen. Der fertige Impfstoff kann per Injektion oder Schluckimpfung verabreicht werden.

COVID-19-Pandemie. Sie zeigt, wozu Forschung mittlerweile in der Lage ist, welche unterschiedlichen Wege dabei gegangen werden können – aber auch, wo sie an ihre Grenzen stößt.

Grundsätzlich stellt sich zunächst die Frage nach der Wahl des geeigneten Pathogen-Antigens; ebenso bedeutsam ist jedoch die Frage nach der Wahl der Technologie – also der Art und Weise, es in den Wirtskörper einzuführen, um dort eine robuste Immunantwort auszulösen. Diese Technologien werden als Plattformtechnologien bezeichnet, die sich nach zwei Konzepten unterscheiden lassen: entweder, das Pathogen-Antigen wird nach Impfung von körpereigenen Zellen hergestellt oder aber es ist selbst Bestandteil des Impfstoffs.

Betrachten wir die Impfstoffentwicklung gegen das SARS-CoV-2 Virus, bei der jeweils das Spike-Protein oder Bestandteile davon als Antigen verwendet wurde [2, 3], fallen die Vakzine auf der Basis von synthetischen Nukleinsäuremolekülen – wie Desoxyribonukleinsäure (DNA) oder Boten-Ribonukleinsäure (mRNA) – in die Kategorie des ersten Konzepts. Beispiele hierfür sind die Impfstoffe der Hersteller BioNTech/Pfizer und Moderna, die die Schnellsten am Markt waren. Ihre Strategie, mRNA-Moleküle zu nutzen, hat zudem erstmals zu einer Zulassung in der Europäischen Union geführt [4, 5].

Alternativ kann das Pathogen-Antigen in Zellkulturen hergestellt werden, um es im Anschluss dem Immunsystem mithilfe unterschiedlicher Technologien zu präsentieren. Werden hierzu ungefährliche Viren verwendet, spricht man von Vektor-Impfstoffen – einen Weg, den die Hersteller AstraZeneca und das Gamaleja-Institut (Entwickler von Sputnik V) bei der Entwicklung gegangen sind. Ist es an eine Trägermatrix gebunden oder wird als separates Protein hergestellt, wird es als Subunit-Vakzin bezeichnet. Hier befinden sich eine ganze Reihe von Kandidaten in der dritten klinischen Phase der Produktentwicklung. Werden hingegen kurze, synthetische Peptide als Antigen verwendet, wie im Impfstoff EpiVacCorona in Russland, handelt es sich um Peptid-Vakzine, die allerdings häufig unzureichende Immunogenität besitzen.

Diese kurze Übersicht zeigt, dass es im Kampf gegen die COVID-19-Pandemie verschiedene vielversprechende Wege gibt, effiziente Impfstoffe zu kreieren. Jedoch bleibt die Problematik, die gesamte Weltbevölkerung möglichst schnell zu immunisieren, um den Wettlauf gegen die Zeit zu gewinnen, eine Herausforderung, die hohe Ansprüche an Produzierbarkeit, Transportfähigkeit, Lagerfähigkeit, Verabreichungsform und Kosten der Impfstoffe stellt – und Probleme verdeutlicht, die weniger wissenschaftlicher als häufig politischer und logistischer Natur sind. Denn hohe Preise, benötigte Kühlketten und ein großer Bedarf an medizinisch geschultem Personal erschweren gerade den Einsatz in weniger technologisch entwickelten Regionen der Erde.

An dieser Stelle setzen wir mit unserem Forschungsansatz an. Das Ziel: eine günstig

Hier steht
eine Anzeige.

Springer

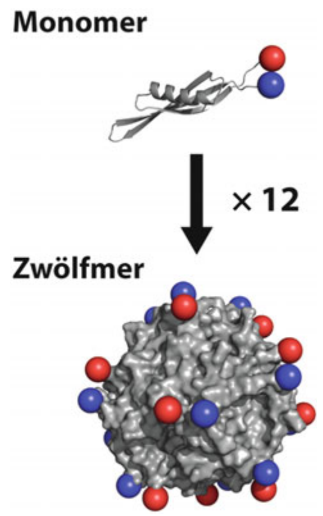

▲ **Abb. 2:** Darstellung der Struktur des Dodecin-Nanopartikels. Die Proteintermini des Dodecins sind als Kugeln hervorgehoben. Rot: N-Terminus; blau: C-Terminus.

zu produzierende und äußerst stabile Trägermatrix für Pathogen-Antigene zu entwickeln, die weder gekühlt noch injiziert werden muss. Der Schlüssel: das Protein Dodecin.

## Das Protein Dodecin – Hoffnung aus einem Tuberkuloseerreger

Jedes Jahr erkranken weltweit etwa zehn Millionen Menschen an Tuberkulose, in rund 1,4 Millionen Fällen führt die Krankheit zum Tod [6] – meist verursacht durch das Bakterium *Mycobacterium tuberculosis*. Doch ausgerechnet in diesem tödlichen Erreger kommt ein Protein vor, das ungewöhnliche und einmalige Eigenschaften besitzt: das Protein Dodecin [7]. Es bildet ein hoch symmetrisches, kugelförmiges Nanopartikel aus, bei dem alle Proteintermini gut zugänglich auf der Oberfläche liegen. Ein Monomer des Zwölfmers ist mit 7–8 Kilodalton (kDa) relativ klein und kann an beiden Enden modifiziert werden. Für die biotechnologische Verwendung sind die Stoffeigenschaften des Proteins von besonderem Interesse: Das Protein ist extrem stabil und toleriert Temperaturen von über 100 °C, stark saures oder basisches Milieu sowie verschiedenste organische Lösungsmittel. Die Zwölfmer-Partikelgröße von etwa 10–15 Nanometer im Durchmesser, potenzielle Fusionen mit eingerechnet, liegt in einem Bereich, der grundsätzlich geeignet ist, eine Immunantwort in Tieren und Menschen zu provozieren [8]. Zusätzlich kann es in großen Mengen rekombinant in Mikroorganismen hergestellt werden, was eine günstige Produktion in Aussicht stellt [7]. All diese Eigenschaften machen Dodecin zu einem vielversprechenden Kandidaten für die Weiterentwicklung zur Trägermatrix für Antigene.

## Vom Protein zum Impfstoff – ein langer Weg

Jeder Impfstoff muss eine Vielzahl von Kriterien erfüllen, um den geimpften Organismus zuverlässig und spezifisch vor einer bestimmten Infektion zu schützen. Im ersten Schritt muss dem Organismus durch die Wahl des richtigen Antigens gezeigt werden, wer „der Feind" ist und wie er zu bekämpfen ist. Diese Information allein reicht jedoch meist nicht aus, um das Immunsystem zu aktivieren, weswegen Adjuvanzien verwendet werden müssen, um die Immunantwort zu steigern. Ziel einer Impfung ist es vor allem, das Gedächtnis des Immunsystems zu trainieren, da unklar ist, zu welchem Zeitpunkt das Pathogen den Wirt erreichen wird – dies kann durch mehrmaliges Verabreichen des Impfstoffs gewährleistet werden. Doch selbst wenn der Impfstoff diese Kriterien erfüllt, bleiben die bereits angesprochenen Probleme in der praktischen Anwendung. Dodecin bietet einige Möglichkeiten, um diese notwendigen Kriterien zu erfüllen und gleichzeitig bestehende Probleme zu lösen.

Nach einem Baukastenprinzip können die Proteintermini variabel mit unterschiedlichen Proteinsequenzen modifiziert werden, ohne dass sich die Grundeigenschaften des Proteins ändern [7]. Diese Modularität wurde in einem Proof-of-Principle-Experiment nachgewiesen, in dem nach Fusion von unterschiedlichen Zielsequenzen spezifische Antikörper gegen diese Sequenzen in Kaninchen erzeugt wurden [7]. Dieses Experiment hat gezeigt, dass die Partikelgröße grundsätzlich ausreichend groß ist, um in Säugetieren eine Immunantwort zu provozieren, es muss jedoch noch gezeigt werden, dass dies auch für den Menschen gilt.

Zur erfolgreichen Weiterentwicklung der Trägermatrix zu einer Plattformtechnologie zur Impfstoffentwicklung ist vor allem die Qualität der stimulierten Immunantwort entscheidend. Die molekulare Immunologie verspricht hier neue Lösungsansätze, die nicht auf der Beimischung von Adjuvanzien basieren, sondern auf der Stimulation bestimmter Rezeptoren mittels Peptid-Liganden. Diese können direkt an Dodecin angehängt werden, sodass sie integraler Bestandteil der Trägermatrix werden. Beispiele hierfür sind die Toll-like-Rezeptor-Agonisten [9] oder aber Anhänge für *M-cell targeting*, welche für orale Vakzine bedeutsam wären. Dodecin bietet hierbei die Möglichkeit, einen Nanopartikel aus verschiedenen Untereinheiten aufzubauen, um in solchen Hetero-Zwölfmeren mehr als zwei Modifikationen zu verankern.

Abschließend stellt die hohe Stabilität des gesamten Nanopartikels eine Lagerung in trockener Form in Aussicht, was die Ansprüche an Transport und Kühlung deutlich reduzieren würde – ein entscheidender Faktor, vor allem für die Verabreichung in Schwellen- und Entwicklungsländern der südlichen Hemisphäre.

## Schluckimpfungen – die Zukunft moderner Vakzine?

Für eine breite Anwendbarkeit in der Veterinärmedizin, aber auch und vor allem für alle Menschen, wird einem modernen Vakzin viel abverlangt. Das hat nicht zuletzt der Kampf gegen die COVID-19-Pandemie eindrucksvoll bewiesen. Hierbei könnte nicht nur Produk-

**Tab. 1:** Eigenschaften des Dodecins und deren Nutzung für die Impstoffentwicklung.

| Dodecin-Nanopartikel | Vorteile in der Anwendung |
|---|---|
| • hohe Stabilität & Säureresistenz | • übersteht potenziell den Magen (orale Vakzine)<br>• Trocknung möglich (einfache Lagerung)<br>• für großtechnische Reinigungsverfahren geeignet |
| • Zwölfmer-Assemblierung weitgehend unbeeinflusst von Fusionen | • Nanopartikel kann einfach modifiziert werden (Baukastenprinzip) |
| • Hetero-Zwölfmere | • polyfunktionale Nanopartikel |
| • gute Exprimierbarkeit in Mikroorganismen | • günstige mikrobielle Produktion |

tion, Transport und Haltbarkeit, sondern auch die Art der Verabreichung eine entscheidende Rolle spielen – und orale Vakzine versprechen eine mögliche Lösungsstrategie. Durch Schluckimpfungen könnte die Impfbereitschaft erhöht und auf medizinisches Personal verzichtet werden. Injektionsbegleitende Risiken durch versehentliches Stechen an Kanülen und damit einhergehendem Bluttransfer würden minimiert, sodass versehentliche Übertragungen mit beispielsweise Hepatitis- oder HI-Virus verhindert werden würden [10].

Durch ihre Eigenschaften, die harschen Bedingungen des Magens unbeschadet zu überstehen, haben Vakzine basierend auf Dodecin das Potenzial, nach Trocknung in Form von Tabletten oder Lösungen verabreicht werden zu können. Eine Plattformtechnologie auf Basis von Dodecin könnte die Entwicklung von Schluckimpfstoffen beschleunigen und deren globale Verfügbarkeit verbessern.

## AUTOREN

Hier steht
eine Anzeige.

Springer


Danksagung

Die Autoren danken Lan-Na Grosse für Ihre Unterstützung beim Verfassen und Korrekturlesen des Texts. M.G. dankt Dieter Oesterhelt für die langjährige Unterstützung. Das Projekt und die Weiterentwicklung der Dodecin plattformtechnologie wird von den Goethe-Corona-Fonds (an M.G.) und dem GO-Bio initial Programm des BMBFs (an F.B. und A.R.) gefördert.

Funding note

Open Access funding enabled and organized by Projekt DEAL.

Florian Bourdeaux 2007–2014 Bachelor- und Masterstudium Chemie an der Universität Frankfurt a. M. Seit 2015 Doktorand bei Prof. Dr. M. Grininger an der Universität Frankfurt a. M. Seit 2020 Projektleiter bei EpiXII (GO-Bio initial Projekt).

Alexander Rittner 2007–2012 Bachelor- und Masterstudium Chemie und Biochemie an der LMU München. 2011 Auslandsaufenthalt zur Masterarbeit bei Prof. Dr. A. Plückthun an der Universität Zürich, Schweiz. 2012–2018 Doktorand bei Prof. Dr. M. Grininger an der Universität Frankfurt a. M. 2018–2020 Postdoc bei Prof. Dr. M. Grininger. Seit 2020 Projektleiter bei EpiXII (GO-Bio initial Projekt).

Martin Grininger: 1995–2002 Studium der Technischen Chemie. 2002–2006 Doktorand am Max-Planck-Institut für Biochemie, Martinsried, bei Prof. Dr. D. Oesterhelt. 2006–2011 Projektgruppenleiter am Max-Planck-Institut für Biochemie. 2012–2019 Lichtenberg-Professor der VolkswagenStiftung an der Universität Frankfurt a. M. Seit 2020 W2-Professor für Biomolekulare Chemie an der Universität Frankfurt a. M.


Literatur

[1] Nourmohammad A Otwinowski J Plotkin JB Hostpathogen coevolution and the emergence of broadly neutralizing antibodies in chronic infections PLOS Genet 2016 12 e1006171 10.1371/journal.pgen.1006171 27442127 PMC4956326
[2] Krammer F SARS-CoV-2 vaccines in development Nature 2020 586 516 527 10.1038/s41586-020-2798-3 32967006
[3] Lan J Ge J Yu J Structure of the SARS-CoV-2 spike receptor-binding domain bound to the ACE2 receptor Nature 2020 581 215 220 10.1038/s41586-020-2180-5 32225176
[4] Sahin U Karikó K Türeci Ö mRNA-based therapeutics — developing a new class of drugs Nat Rev Drug Discov 2014 13 759 780 10.1038/nrd4278 25233993
[5] Cavaleri M Enzmann H Straus S Cooke E The European Medicines Agency’s EU conditional marketing authorisations for COVID-19 vaccines Lancet 2021 397 355 357 10.1016/S0140-6736(21)00085-4 33453149 PMC7833511
[6] World Health Organization (2020). Global tuberculosis report 2020. https://www.who.int/publications/i/item/9789240013131
[7] Bourdeaux F Kopp Y Lautenschläger J Dodecin as carrier protein for immunizations and bioengineering applications Sci Rep 2020 10 13297 10.1038/s41598-020-69990-0 32764653 PMC7414021
[8] Bachmann MF Jennings GT Vaccine delivery: a matter of size, geometry, kinetics and molecular patterns Nat Rev Immunol 2010 10 787 796 10.1038/nri2868 20948547
[9] Kumar S Sunagar R Gosselin E Bacterial protein toll-like-receptor agonists: a novel perspective on vaccine adjuvants Front Immunol 2019 10 1144 10.3389/fimmu.2019.01144 31191528 PMC6549121
[10] Vela Ramirez JE Sharpe LA Peppas NA Current state and challenges in developing oral vaccines Adv Drug Deliv Rev 2017 114 116 131 10.1016/j.addr.2017.04.008 28438674 PMC6132247
